# Dr. Nathan Smith Lincoln

**Published:** 1898-11

**Authors:** 


Dr. Nathan Smith Lincoln, of Washington, D. C., died at his residence in that city October 14, 1898, aged 70 years. Dr. Lincoln was one of the foremost surgeons of his day, and during the civil war served as surgeon-in-chief of the quartermaster hospitals at Washington. He will be remembered as one of the surgeons who attended President Garfield after the assassination. He was the last but one of that group to survive. Dr. Robert Reyburn is the other. Dr. Lincoln was an able physician, a scholarly man and a good citizen.

